# Reduced BMP Signaling Results in Hindlimb Fusion with Lethal Pelvic/Urogenital Organ Aplasia: A New Mouse Model of Sirenomelia

**DOI:** 10.1371/journal.pone.0043453

**Published:** 2012-09-17

**Authors:** Kentaro Suzuki, Yasuha Adachi, Tomokazu Numata, Shoko Nakada, Motoko Yanagita, Naomi Nakagata, Sylvia M. Evans, Daniel Graf, Aris Economides, Ryuma Haraguchi, Anne M. Moon, Gen Yamada

**Affiliations:** 1 Department of Development of Genetics, Institute of Advanced Medicine, Wakayama Medical University (WMU), Kimiidera, Wakayama, Japan; 2 Department of Organ Formation, Institute of Molecular Embryology and Genetics (IMEG), Kumamoto University, Kumamoto, Japan; 3 Graduate School of Medicine, Kyoto University, Kyoto, Japan; 4 Division of Reproductive Engineering, Center for Animal Resources and Development, Kumamoto University, Kumamoto, Japan; 5 Skaggs School of Pharmacy and Pharmaceutical Sciences, University of California San Diego, La Jolla, California, United States of America; 6 Department of Medicine, University of California San Diego, La Jolla, California, United States of America; 7 Institute of Oral Biology, Faculty of Medicine, University of Zurich, Zurich, Switzerland; 8 Genome Engineering Technologies, Regeneron Pharmaceuticals, Tarrytown, New York, United States of America; 9 Department of Molecular Pathology, Ehime University Graduate School of Medicine, Ehime, Japan; 10 Weis Center for Research, Geisinger Clinic, Danville, Pennsylvania, United States of America; Children's Hospital Los Angeles, United States of America

## Abstract

Sirenomelia, also known as mermaid syndrome, is a developmental malformation of the caudal body characterized by leg fusion and associated anomalies of pelvic/urogenital organs including bladder, kidney, rectum and external genitalia. Most affected infants are stillborn, and the few born alive rarely survive beyond the neonatal period. Despite the many clinical studies of sirenomelia in humans, little is known about the pathogenic developmental mechanisms that cause the complex array of phenotypes observed. Here, we provide new evidences that reduced BMP (Bone Morphogenetic Protein) signaling disrupts caudal body formation in mice and phenocopies sirenomelia. *Bmp4* is strongly expressed in the developing caudal body structures including the peri-cloacal region and hindlimb field. In order to address the function of *Bmp4* in caudal body formation, we utilized a conditional *Bmp4* mouse allele (Bmp4^flox/flox^) and the *Isl1 (Islet1)*-Cre mouse line. *Isl1*-Cre is expressed in the peri-cloacal region and the developing hindimb field. *Isl1Cre;Bmp4^flox/flox^* conditional mutant mice displayed sirenomelia phenotypes including hindlimb fusion and pelvic/urogenital organ dysgenesis. Genetic lineage analyses indicate that *Isl1*-expressing cells contribute to both the aPCM (anterior Peri-Cloacal Mesenchyme) and the hindlimb bud. We show *Bmp4* is essential for the aPCM formation independently with *Shh* signaling. Furthermore, we show *Bmp4* is a major BMP ligand for caudal body formation as shown by compound genetic analyses of *Bmp4* and *Bmp7*. Taken together, this study reveals coordinated development of caudal body structures including pelvic/urogenital organs and hindlimb orchestrated by BMP signaling in *Isl1*-expressing cells. Our study offers new insights into the pathogenesis of sirenomelia.

## Introduction

Sirenomelia, also known as mermaid syndrome, is a developmental malformation characterized by leg fusion [1]. The incidence of sirenomelia is reported as approximately 1 in 100,000 births [2]. In addition to leg fusion phenotypes, sirenomelia presents with abnormal pelvic/urogenital organ development affecting the bladder, kidney, rectum and external genitalia. This constellation of phenotypes suggests that development of the caudal body such as hindlimb and pelvic organs is coordinated. Due to the striking leg fusion phenotypes in sirenomelia, most researchers have focused on understanding the pathogenesis of this feature. Clinical and anatomical studies in humans have resulted in two hypotheses for the pathogenesis of sirenomelia: vascular steal and a defect of blastogenesis. The vascular steal hypothesis posits that hindlimb fusion results from deficient blood flow and nutrient supply to the caudal mesoderm, while a primary anomaly in the process of the caudal mesoderm formation is attributed by defective blastogenesis [1], [3]–[5]. However, the molecular and developmental bases of the complex phenotypes have not been elucidated.

During mouse development, a transient embryonic cavity, the cloaca, forms at the caudal end of the hindgut and is subsequently divided into the urogenital sinus and the rectum by the urorectal septum (URS) [6]–[10]. The bladder and rectum are pelvic organs derived from the cloaca. Recently, we showed that the Peri-Cloacal Mesenchyme (PCM) is another essential tissue for development of the pelvic organs and external genitalia [11], [12]. Previous study describes the PCM as infra-umbilical mesenchyme [13]. PCM region has been originally suggested as part of the *Gli1*, an indicator gene of hedgehog (HH) signaling, positive mesenchyme [11]. Previous lineage analysis suggests that PCM also includes the anterior part of cloaca [11]. To precisely define and discuss its role, the anterior part of the PCM region is hereafter designated as aPCM (anterior Peri-Cloacal Mesenchyme).

*Shh*, a major ligand of HH signaling for the aPCM formation, is expressed also in the anterior part of cloaca. *Shh* KO mice show the abnormal development of the aPCM-derived tissues such as external genitalia [14]. In addition to the roles of *Shh* in aPCM-derived tissue formation, the significance of the aPCM region to coordinate caudal organ formation is unknown.

Bone Morphogenetic Protein (BMP) signaling regulates a range of cellular processes and plays essential roles regulating the morphogenesis of many organs [15]–[19]. BMPs are members of the evolutionarily conserved Transforming growth factor-β (TGF-β) superfamily that signals via type I and type II receptors. *Bmp7* KO mice die shortly after birth and display defects of kidney development [20], [21]. Although *Bmp7* KO mice do not have defective caudal body formation, ablation of both *Bmp7* and *Twsg1* (which encodes a modulator of BMP signaling) results in hindlimb fusion [22]. Due to the functional redundancy of BMP genes, little is known about the role of individual BMPs in caudal body development. Therefore, it is necessary to address the role of BMP signaling using compound genetic analyses of *Bmp* mutant alleles. Intriguingly, *Bmp4* is expressed in the caudal body region including the anterior cloacal mesenchyme (anterior mesenchyme adjacent to the cloaca before the formation of aPCM) and hindlimb field. However, *Bmp4*-null embryos die between embryonic day (E) 6.5 and E9.5, prior to caudal body formation [23]. Thus, determining the function of *Bmp4* in the caudal body requires a conditional gene ablation approach.

A subset of hindlimb progenitors have been identified by fate mapping studies with an *Isl1 (Islet1)*-Cre mouse line generated by a knock-in of the Cre gene into the endogenous *Isl1* locus [24]. *Isl1*, a LIM-homeodomain-transcription factor regulates the process of hindlimb initiation [25]. *Isl1*-Cre is expressed not only in the hindlimb field, but also in the cloacal regions [24]. We observed that *Isl1*-expressing cells contribute to the aPCM and subsequently to the caudal body structures including the bladder, rectum and external genitalia. To investigate the role of *Bmp4* during caudal body formation, we analyzed the *Bmp4* conditional KO mice utilizing *Isl1*-Cre driver strain. These *Bmp4* conditional KO mice showed sirenomelia phenotypes including hindlimb fusion and also hitherto undescribed lethal pelvic/urogenital organ dysplasia. We show that *Bmp4* is required to form the aPCM and to adjust hindlimb positioning during caudal body formation. Our study revealed a novel requirement of *Bmp4* function and an essential population of progenitor cells for caudal body formation.

## Results

### Disruption of *Bmp4* function leads to the sirenomelia

**Figure 1:**
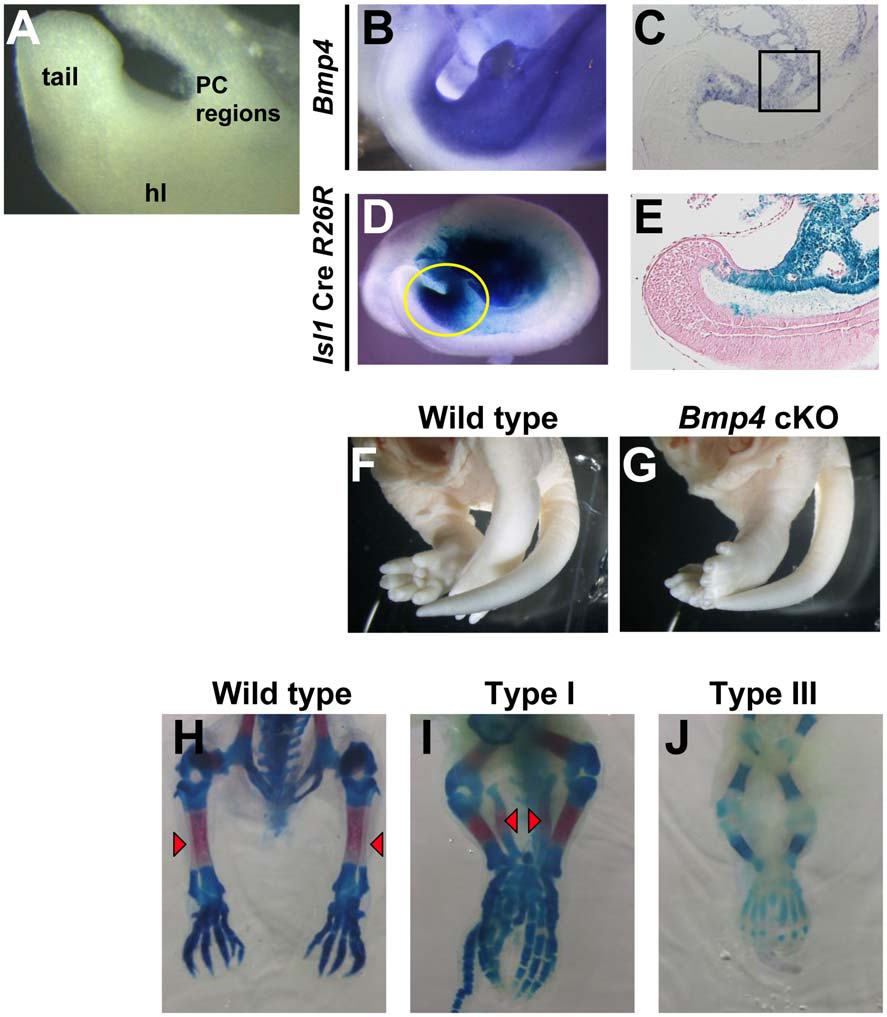
Hindlimb fusion of *Bmp4* cKO mice. (A) Diagram of peri-cloacal regions. Peri-cloacal regions (PC-regions) including the base of the umbilical cord and anterior cloacal mesenchyme. hl, hindlimb field. (B, C) In situ hybridization (ISH) analysis reveals *Bmp4* expression in the peri-cloacal regions (square in C) and hindlimb field at E9.5. (D, E) The *R26R-lacZ* Cre reporter shows that the *Isl1*-Cre expressing cells are present in the peri-cloacal regions and the developing hindimb field at E9.5 (D, yellow circle, E). (F–J) Hindlimb fusion shown by gross appearance and by skeletal preparations of *Bmp4* cKO mice. The fibulae are aberrantly located medially (I) or are absent (J) in the mutants. Red arrowheads indicate the ossified fibula.

We found that *Bmp4* is strongly expressed in the caudal tissues including the base of the umbilical cord and anterior cloacal mesenchyme (hereafter designated as peri-cloacal regions) and the hindlimb field at early staged embryo of E9.5 (Fig. 1 A, B and C, square). In order to address the function of *Bmp4* in caudal body development, we utilized a conditional *Bmp4* null allele (Bmp4^flox/flox^) and the *Isl1*-Cre mouse line [24]. *Isl1*-Cre is expressed in the caudal body regions, including the peri-cloacal regions and the mesenchyme of the developing hindimb field at E9.5 (Fig. 1 D, yellow circle, E) [24]. *Isl1Cre;Bmp4^flox/flox^* (hereafter designated as *Bmp4* cKO) mutants possess hindlimb fusion similar to sirenomelia in humans (Fig. 1 G). Stocker and Heifetz classified sirenomelia into types I-VII, based on which skeletal elements are present and their relationship within the malformed extremity [26]. We therefore analyzed hindlimb skeletons of 22 mutant mice (Fig. 1 H–J). Based on the variants described by Stocker and Heifetz, 59% and 36% of the mutants had defects consistent with type III: loss of the fibula, and type I: abnormal medial location of fibula, respectively. The other 5% had type V sirenomelia limb phenotypes: loss of the fibula and fusion of the femur (Fig. 1 H–J, red arrowheads indicate the position of the ossified fibula).

**Figure 2:**
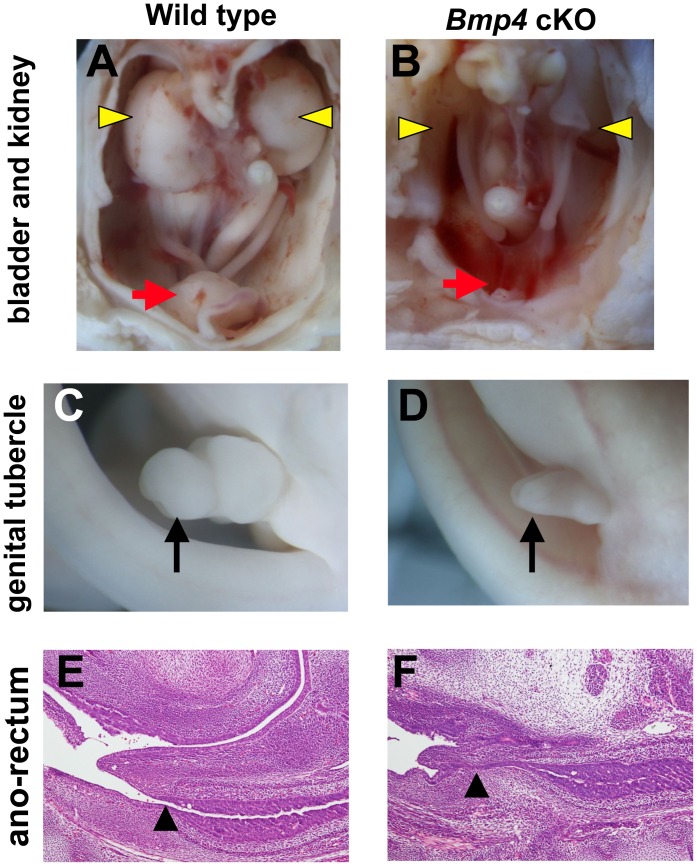
Defective formation of the pelvic/urogenital organs in *Bmp4* cKO mice. (A–F) *Bmp4* cKO mice have bladder aplasia (red arrow in B) and hypoplastic kidney (yellow arrowheads in B), hypoplasia of external genitalia (arrow in D) and anal stenosis (arrowhead in F). red arrow, bladder; yellow arrowhead, kidney; arrow, genital tubercle; arrowhead, ano-rectal region.

Anomalies in the pelvic/urogenital organs are the major cause of lethality in human sirenomelia patients. Thus, we analyzed the pelvic/urogenital organs of *Bmp4* cKO mice. *Bmp4* cKO mice had bladder aplasia, hypoplastic kidney, hypoplasia of external genitalia and anal stenosis (Fig. 2 B, D, F). These results suggest that disruption of *Bmp4* function in caudal body regions phenocopies all prominent phenotypes of sirenomelia observed in humans.

### Genetic analysis for tissue contribution of *Isl1*-expressing cells to the embryonic caudal body

**Figure 3:**
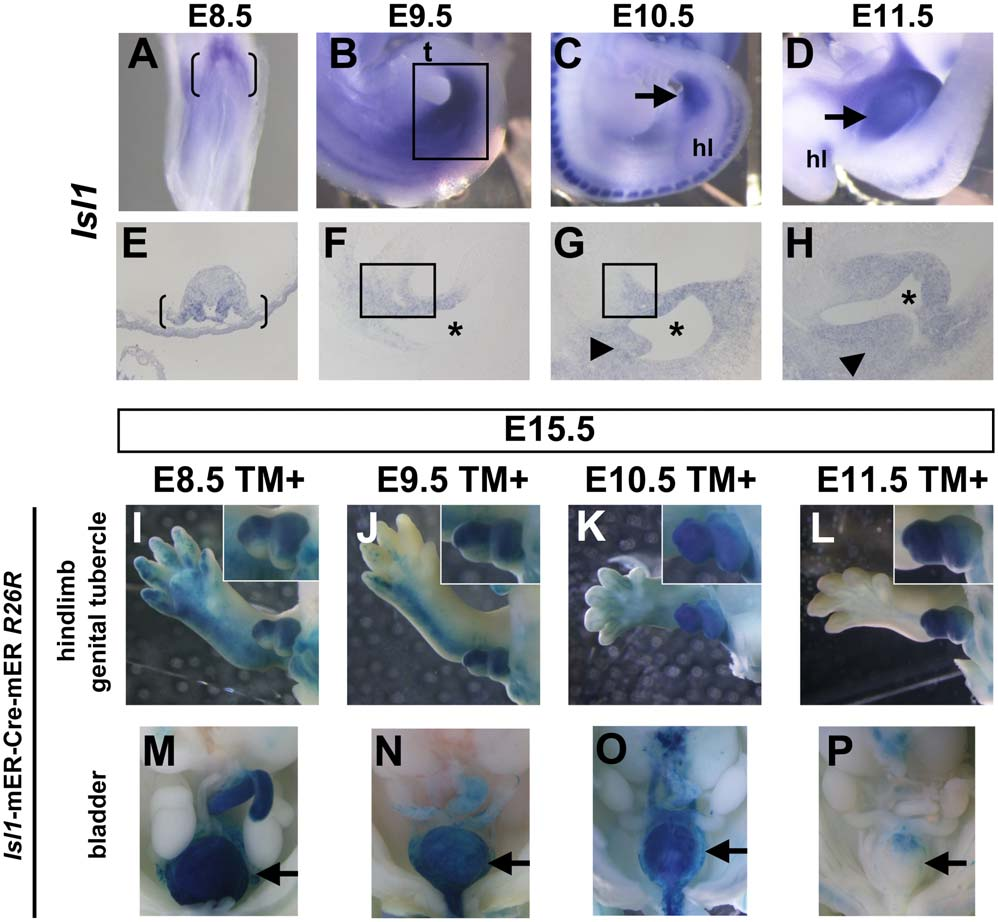
Tissue contribution of *Isl1*-expressing cells to the caudal body. (A–H) Expression pattern of *Isl1* mRNA during caudal body development. (A, E) *Isl1* is expressed in the lateral plate mesoderm adjacent to the future hindlimb bud and the base of the allantois at E8.5 (bracket in A and E). (B, F) *Isl1* is expressed in the caudal body region and hindlimb bud at E9.5 (square in B). Its expression is detected in the peri-cloacal regions (square in F). (C, G) *Isl1* expression is detected in the cloacal region at E10.5 (arrow in C). It is expressed in the URS (arrowhead in G) and cloacal mesenchyme including aPCM (square in G). *Isl1* expression in the hindlimb bud is reduced at E10.5. (D, H) Its expression is maintained in the developing GT at E11.5 (arrow in D). Its expression is detected in GT mesenchyme and URS (arrowhead in H). Asterisk indicates cloaca. (I–P) The *R26R-lacZ* Cre reporter shows LacZ staining of caudal body regions in *Isl1*-mER-Cre-mER embryos at E15.5 after administration of tamoxifen at E8.5–E11.5. Whole-mount view of stained embryos of hindlimb and external genitalia (I–L) and pelvic organs (M–P). *Isl1*-expressing cells contribute to the hindlimb, external genitalia and bladder. Insets in I–L are high magnification of GT. Ventral GT is located at the bottom. t, tail; hl, hindlimb bud.

The above results prompted us to genetically analyze tissues contributing to caudal body formation with regard to *Isl1* and *Bmp4* expression domains. *Isl1* mRNA was observed in the lateral plate mesoderm adjacent to the future hindlimb bud and at the base of the allantois at E8.5 (Fig. 3 A, E, bracket). Its expression was detected in the peri-cloacal regions (the base of the umbilical cord and anterior cloacal mesenchyme) and the hindlimb field at E9.5 (Fig. 3 B, F, square). *Isl1* was expressed in the cloacal mesenchyme including aPCM, and URS (urorectal septum) at E10.5 (Fig. 3 C, arrow, G, square and arrowhead). Its expression in the hindlimb bud was decreased after E10.5, but maintained in the developing genital tubercle, an anlage of external genitalia, (hereafter designated as GT) at E11.5 (Fig. 3 D, arrow, H).

**Figure 4:**
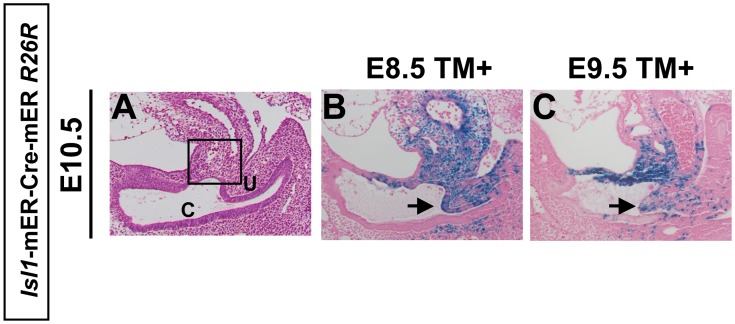
*Isl1*-expressing cells contribute to the aPCM. (A–C) Mid-sagittal sections of cloacal region at E10.5. The square indicates the aPCM (A). The *Isl1*-expressing cells contribute to the aPCM and URS (B, C). Arrows indicate the URS. u, URS; c,cloaca.

Previous fate mapping with *Isl1*-Cre and a *R26R*-*lacZ* reporter mouse strain (*R26R*) [27], revealed that *Isl1*-expressing progenitors contribute to the hindlimb field [24]. In order to address the contribution of *Isl1*-expressing cells during caudal body formation, we used the *Isl1*-mER-Cre-mER allele in which sequences encoding a tamoxifen-dependent Cre recombinase were inserted into the *Isl1* locus [28]. In order to identify *Isl1*-expressing cells at each embryonic stage, tamoxifen (hereafter designated as TM) was administered between E8.5 and E11.5 and the resulting β-galactosidase labeled cells in *Isl1*-mER-Cre-mER;*R26R* embryos were analyzed at E15.5 (Fig. 3 I–P). The TM-inducible labeling system marks cells within approximately less than a half day of TM administration [29], [30]. We observed that the majority of *Isl1*-expressing cells contribute to the posterior hindlimb (Fig. 3 I, J), dorsal GT (Fig. 3 I, J, inset) and the bladder (Fig. 3 M, N, arrow) with TM induction at E8.5 and E9.5. Induction at E10.5 and E11.5 resulted in labeled cells in both dorsal and ventral GT (Fig. 3 K, L, inset), bladder (Fig. 3 O, P, arrow), and in a restricted portion of the hindlimb (Fig. 3 K, L). TM induction at E11.5 labeled only some cells in the apical region of the bladder (Fig. 3 P). Previously, we reported that aPCM (anterior Peri-Cloacal Mesenchyme) contributes to the dorsal GT and the mesenchyme of bladder [11]. The aPCM region has been originally described as part of PCM, which is located in the mesenchyme surrounding the anterior part of cloaca [11]. These observations prompted us to examine whether *Isl1*-expressing cells contribute to the aPCM formation. Thus we induced with TM at E8.5 and E9.5 and analyzed the LacZ labeled cells in *Isl1*-mER-Cre-mER;*R26R* embryos at E10.5. Predictably, *Isl1*-expressing cells were located in the aPCM at E10.5 (Fig. 4 B, C). Furthermore, we detected the labeled cells in the URS (urorectal septum) (Fig. 4 B, C, arrow). These results suggest that *Isl1*-expressing cells (between E8.5 and E9.5) contribute to the pelvic/urogenital organs, external genitalia, and hindlimb formations. Taken together, coordinated formation of caudal body may be orchestrated by *Isl1*-expressing cells.

### Abnormal aPCM formation of *Bmp4* cKO mice

**Figure 5:**
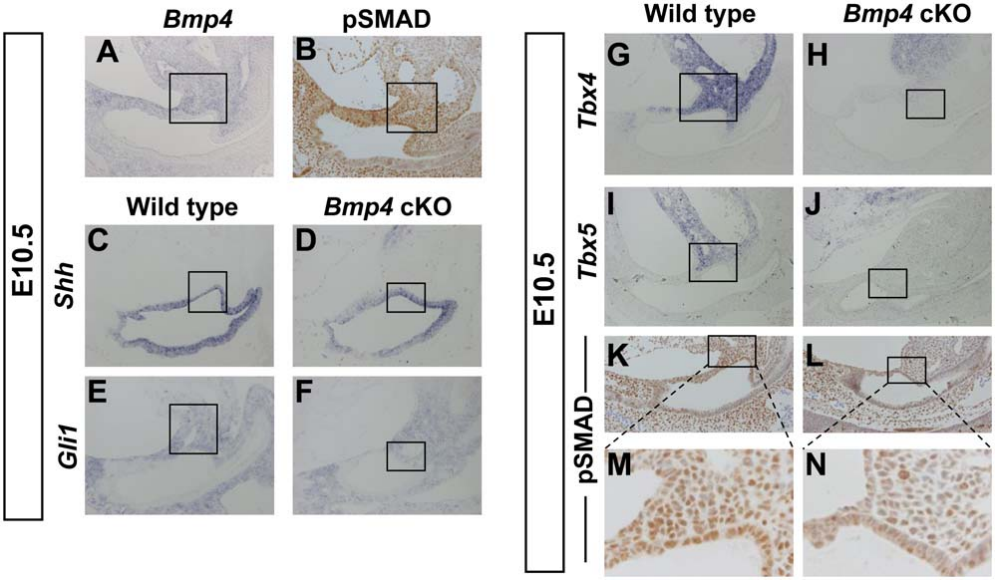
Defective aPCM formation of *Bmp4* cKO mice. (A) *Bmp4* is expressed in the aPCM at E10.5. (B) Immunohistochemical analysis of pSMAD in the aPCM at E10.5. (C–J) Section in situ hybridization analysis with the aPCM and cloacal marker genes for wild type (C, E, G, I) and mutant embryos (D, F, H, J) at E10.5. (K–N) Immunohistochemical analysis of pSMAD in the aPCM of wild types (K) and mutant embryos (L) at E10.5. (M, N) High magnification images of square region in K and L. The squares in A–L indicate the aPCM.

We observed that *Bmp4* was expressed in the peri-cloacal regions at E9.5 and thereafter in the aPCM at E10.5 (Fig. 1 C, Fig. 5 A). We also observed active BMP signaling in the aPCM based on anti-pSMAD immunohistochemical analysis (Fig. 5 B). This led us to investigate whether *Bmp4* regulates the aPCM formation and to analyze expression of cloacal and aPCM marker genes (*Shh, Gli1, Tbx4 and Tbx5*) in *Bmp4* cKO mice. HH (Hedgehog)-responding cells contribute to both the dorsal GT and the mesenchyme of bladder [11]. Of note, the expression of *Shh*, a critical ligand of HH signaling for the aPCM formation, was not altered in the cloacal endoderm of *Bmp4* cKO mice (Fig. 5 C, D). *Gli1* is an indicator of HH signaling and is expressed in the aPCM and mesenchyme of the URS (Fig. 5 E) [11]. *Gli1* was expressed in these regions of *Bmp4* cKO mice (Fig. 5 F). *Tbx4* and *Tbx5* are developmental genes for limb initiation and outgrowth. They were normally expressed in the aPCM of wild type (Fig. 5 G, I), but such expression was dramatically reduced in *Bmp4* cKO mice (Fig. 5 H, J). In contrast, these expression remained in *Shh* KO mice (Fig. S1). Contrary to the case of aPCM, the *Bmp4* cKO mice showed sustained expression of *Tbx4* and *Tbx5* in the limb bud (data not shown). BMP signaling was reduced in the mutant aPCM region based on pSMAD immunoreactivity (Fig. 5 K–N).

It has been shown that *Isl1* is essential for hindlimb initiation [25]. Although *Isl1* is expressed in the aPCM and URS at E10.5 (Fig. 3 G), the function of *Isl1* in the development of pelvic/urogenital organs has not been examined. Therefore, we analyzed the *Isl1* conditional KO mice during caudal body formation. We utilized *Hoxa3*-Cre, which is expressed broadly in the caudal body region from E8.5 [31]. The mutant embryos showed hypoplastic aPCM and defective hindlimb initiation, but the expression of *Gli1* and *Bmp4* persisted in the aPCM (data not shown). Taken together, these results show that BMP4 signaling in the caudal *Isl1* expression domains is required for the aPCM formation and loss of this signal (rather than decreased *Isl1* function) causes the profound phenotypes of *Isl1Cre;Bmp4^flox/flox^* mutants.

**Figure 6:**
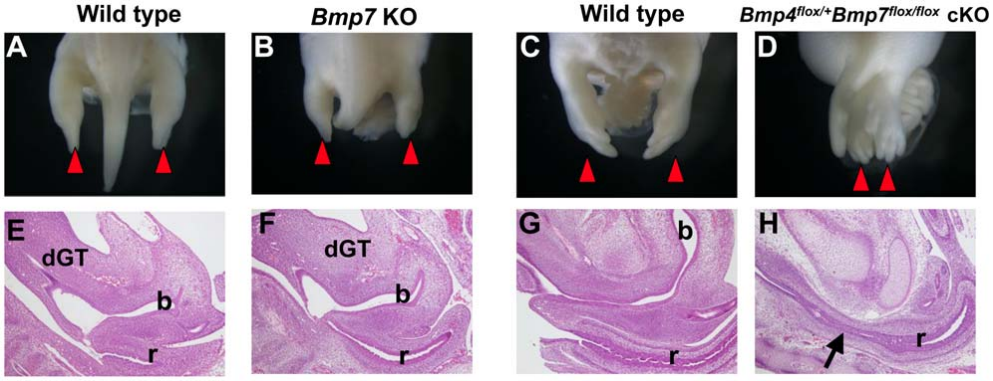
Genetic interaction between *Bmp4* and *Bmp7* during the caudal body formation. (A–D) Whole-mount view of wild type and mutant embryonic hindlimb. Red arrowheads indicate the hindlimb position. (E–H) Mid-sagittal sections of wild type and mutant embryos. *Bmp7* KO mice do not show hindlimb fusion and prominent bladder defects at E13.5 (B, F). *Hoxa3*-Cre;*Bmp4^flox/+^Bmp7^flox/flox^* double compound mutant mice show the hindlimb fusion at E14.5 (D). The compound mutant mice also show bladder aplasia (H) and anal stenosis (arrow in H). dGT, dorsal GT; b, bladder; r, rectum.

Functional redundancy of BMP genes has been shown in multiple developing organ systems [32]–[35]. *Bmp4* cKO mice have both hindlimb fusion and defective pelvic/urogenital organs indicating that BMP4 is the critical ligand for caudal body development. *Bmp7* KO mice do not show sirenomelia phenotypes (Fig. 6 B, F), but display kidney hypoplasia [20], [21]; implying possible functional redundancy of BMP signaling. Indeed, loss of a single copy of *Bmp4* and both copies of *Bmp7* in *Bmp4^flox/+^Bmp7^flox/flox^* compound mutant mice (*Hoxa3*-Cre;*Bmp4^flox/+^Bmp7^flox/flox^*) resulted in hindlimb fusion (Fig. 6 D) as well as loss of the bladder and anal stenosis, essential diagnotic features of sirenomelia (n = 3) (Fig. 6 H). The same phenotypes were also observed in such double compound mutant mice. These results indicate that *Bmp4* compensates for the loss of *Bmp7* during caudal body development.

### Developmental patterning of hindlimb bud in *Bmp4* cKO mice

**Figure 7:**
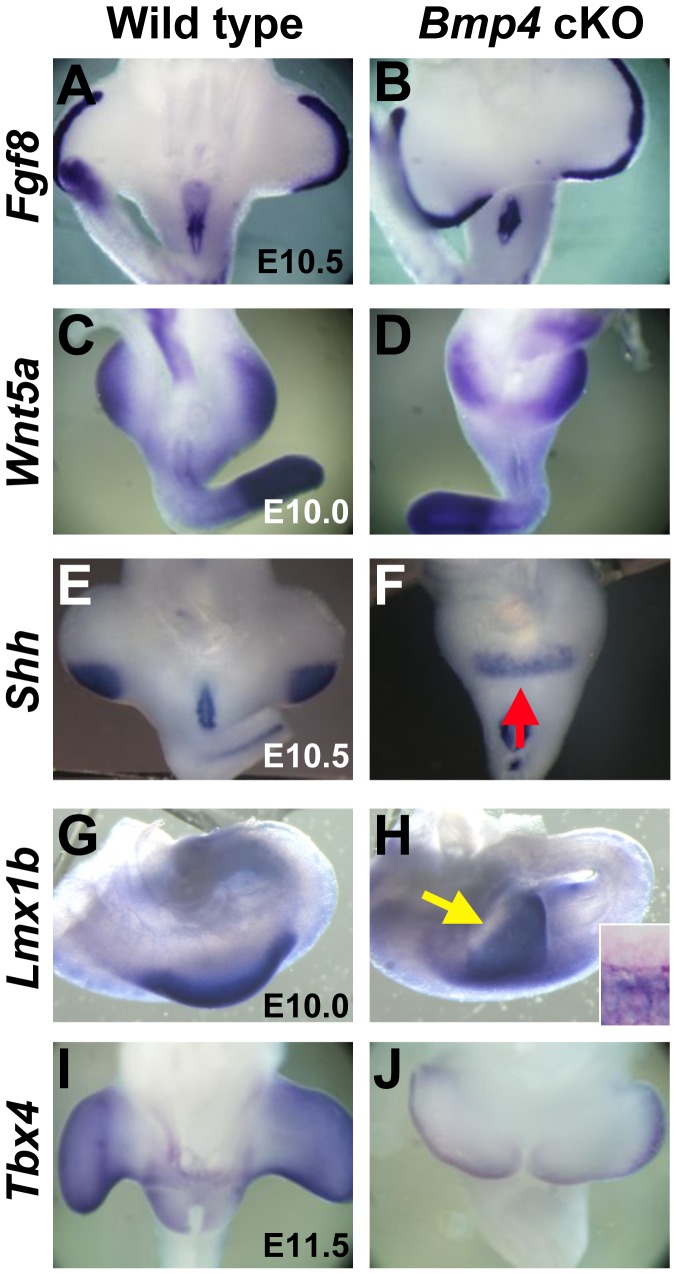
Developmental patterning of hindlimb bud in *Bmp4* cKO mice. (A–J) ISH analysis with the limb patterning genes for wild type and mutant embryos. Marker genes are indicated in the left of the panels.

Sirenomelia patients possess characteristic leg phenotypes including the fusion, abnormal medial rotation and defective skeletal formation of fibula [26]. Pattern formation is an essential process for hindlimb development. Limb patterning is established along three axes: proximal-distal (P-D: hip to toe), anterior–posterior (A–P: 1^st^ to 5^th^ digit; tibia to fibula) and dorsal–ventral (D–V: top versus plantar foot). To analyze the patterning of hindlimb in *Bmp4* cKO mice, we performed gene marker analyses of the hindlimb bud. P-D limb development depends on a specialized epithelium at the distal tip of the limb bud, the apical ectodermal ridge (AER) [36]–[39]. Although *Fgf8*, the essential AER marker, was expressed normally in the AER, the location of hindlimb bud was closely apposed to one another in the *Bmp4* cKO mice (Fig. 7 A, B). *Wnt5a* is expressed in the distal mesenchyme and essential for the proximo-distal limb outgrowth [40] (Fig. 7 C). *Wnt5a* expression was detected in the mutant mice in a bowl-shaped pattern at the ventral-caudal midline of the body (Fig. 7 D). The findings on *Fgf8* and *Wnt5a* expression indicate that the P-D axis of the hindlimb bud is maintained in *Bmp4* cKO mice. *Shh* is expressed in the posterior limb bud regulating the A-P patterning of the limb [41]–[43]. *Shh* was expressed in the mesenchyme of posterior limb bud of wild type (Fig. 7 E). *Bmp4* cKO mice showed shifted location of *Shh* expression in the midline of the caudal body (Fig. 7 F, red arrow). These observations suggest that posterior hindlimb buds are fused in the *Bmp4* cKO mice. However, there were some phenotype variations of *Shh* expression in the mutants. Some mutants showed the prominent reduction of its expression in the hindlimb bud (data not shown). In accordance with such variable expression pattern, digit phenotype was also variable (data not shown). In fact, several papers suggest various digit phenotypes in human patients of sirenomelia [44], [45]. The transcription factor *Lmx1b* is expressed in the dorsal mesenchyme of the limb bud and is a primary regulator of dorsal limb identity [46], [47]. Its expression was detected in the mesenchyme of mutant mice at the ventral caudal midline of the body (Fig. 7 H, yellow arrow and inset). These results suggest that the hindlimb patterning is basically maintained in *Bmp4* cKO mice. Taken together, these results suggest early hindlimb bud grows out normally but rather the midline tissue is missing. Thus the hindlimbs may be fused and abnormally rotated. Abnormalities in the aPCM of *Bmp4* cKO mice may lead to such loss of midline structure in the mutant mice subsequently leading to the approximation of hindlimb to the midline with change of the *Lmx1b* expression. Recent reports indicate that *Tbx4* regulates the formation of hindlimb skeletal elements such as fibula [48], [49]. Expression of *Tbx4* was reduced in *Bmp4* cKO mice (Fig. 7 I, J). These results suggest a possibility that the defective patterning of *Tbx4* expression may cause the abnormal fibula formation in the mutant embryos.

## Discussion

### *Bmp4* cKO mice as mouse model of sirenomelia with hindlimb fusion and lethal pelvic/urogenital organ aplasia

We have identified *Bmp4* cKO mice as a new mouse model for sirenomelia. Unlike previous mouse models, the current model displays all the key phenotypes of sirenomelia including hindlimb fusion, dysgenesis of pelvic/urogenital organs and hypoplasia of external genitalia. The kidneys and upper urinary tract are retro-peritoneal organs whereas the bladder and urethra are caudal intra-peritoneal organs derived from the cloaca. Our study has revealed a surprisingly wide range of abnormalities affecting the external genitalia as well as intra-pelvic organs and retro-peritoneal organs.

Current tissue lineage analyses suggest that *Isl1*-expressing cells are essential population of cells for the caudal body formation including pelvic/urogenital organs and hindlimb. We found *Isl1*-expressing cells contribute to the aPCM formation. *Tbx4* and *Tbx5* are expressed in the aPCM region. *Tbx4* is expressed in the umbilical cord, aPCM and also hindlimb bud. On the other hand, *Tbx5* is not expressed in the hindlimb bud and its expression is more restricted in the aPCM. Thus, *Tbx5* would be a one of the appropriate markers for the aPCM. *Bmp4* cKO mice show defective tissue formation such as bladder agenesis and GT hypoplasia, which are derived from the aPCM with reduced *Tbx4* and *Tbx5* expression. *Tbx4*-expressing cells contribute to the mesenchyme of the bladder and GT by lineage analysis with *Tbx4*-Cre *R26R* mice [50]. These results suggest that the aPCM is not formed in *Bmp4* cKO mice. Hence, current observations indicate that the aPCM contains essential progenitors for pelvic/urogenital tissues. Normally, *Bmp4* is expressed and BMP signaling is active in the aPCM during caudal body formation. Loss of pSMAD immunoreactivity in the aPCM region of *Bmp4* cKO mice indicates that autocrine BMP4 action is required to form the aPCM. To investigate the role of BMP4 in the aPCM formation, we assessed apoptosis and cell proliferation by Tunel and EdU assay, respectively. Both conditions were not altered in *Bmp4* cKO mice (data not shown). It has been shown that BMP signaling regulates the supply of mesenchymal cells during gastrulation [51]. Elucidation of another signaling crosstalks with BMP in the developing caudal body will greatly facilitate our understanding of molecular pathogenesis of sirenomelia. Further analysis is necessary to understand the function of BMP4 for the formation of the aPCM.

The current study indicates *Bmp4* in the *Isl1*-expressing cells contribute not only to the aPCM but also in the URS formation. Cloacal septation by the URS is an essential process for the anorectal formation [9]. Previous study shows that *Shh* signaling is also essential for the anorectal formation. The expression of *Shh* in the endoderm was remained in *Bmp4* cKO mice. These results indicate that BMP signaling is involved for the aPCM formation independently with *Shh* signaling.

An essentially important pathological feature of sirenomelia is leg fusion. The leg locates in the lateral body wall, which is supported by pelvic skeletons. Previous studies suggest abnormal formation of the leg is derived from the defective midline formation [52], [53]. The current phenotypes are associated with the aplasia of midline structure which is derived from the aPCM region. Although *Shh* KO mice show defective pelvic/urogenital formation, their mutants do not show the hindlimb fusion [14], [54]. Of note, the expression of the aPCM marker genes such as *Tbx4* and *Tbx5* remained in *Shh* KO mice. These observations suggest that proper formation of the aPCM is an essential process for regulating the location of hindlimb development. Although hindlimb bud was located at the ventral caudal midline of the body, the early pattern formation of hindlimb bud was basically not affected in the *Bmp4* cKO mice. Taken together, the current results suggest that proper formation of the aPCM by BMP signaling may be an essential process not only for the formation of pelvic/urogenital organs but also for the determination/allocation of hindlimbs bilaterally in the caudal embryos. In this aspect, the ventral midline of the lower body region between bilateral hindlimb bud develops coordinately with hindlimbs.

It has been reported that *Isl1* is expressed in the caudal lateral plate mesoderm during the caudal body development [55]. *Isl1*-expressing cells contribute not only to the pelvic/urogenital organs but also to hindlimb. One of the classifications of sirenomelia is the defect of skeletal elements in the leg such as fibula [26]. The limb is derived from two tissues sources, the lateral plate mesoderm and somatic cells. The lateral plate mesoderm of the hindlimb field gives rise to the cartilage, bone, perichondrium, tendons, ligaments, and connective tissues. In contrast, muscle and blood vessels arise from somatic cells that migrate into the limb [56]. *Tbx4*-expressing cells contribute to the lateral plate-derived tissues, particularly to the bone, tendon, and perichondrium [50]. *Tbx4* plays a role in formation of the skeletal elements of the limb such as fibula [48]. In the current study, *Bmp4* cKO mice display the defective fibula formation with reduced *Tbx4* expression. The decrease of *Tbx4* expression in the hindlimb bud by defective BMP4 signaling could be a one of the causative factors for the defective skeletal elements in sirenomelia.

### *Bmp4* is a major BMP ligand for the caudal body formation

BMP genes are broadly expressed during embryonic development in a redundant manner [32], [33]. Furthermore, different degree of BMP signaling is required to regulate distinct developmental programs. The current compound genetic analyses of *Bmp4* and *Bmp7* have suggested a redundancy between BMP genes during caudal development. *Bmp7* single KO mice do not show the sirenomelia phenotypes. However, *Bmp7/Twsg1* double compound mutants display the hindlimb fusion like a sirenomelia [22]. *Twsg1* is a one of the modulators of BMP signaling and can act both to promote and inhibit BMP activities. Such observations imply that other BMPs play major roles for the caudal body formation. In the current study, *Bmp4* heterozygous and *Bmp7* homozygous double compound mutant mice, *Hoxa3*-Cre;*Bmp4^flox/+^Bmp7^flox/flox^*, showed not only the hindlimb fusion but also a loss of bladder and anorectal malformations similar to the case of *Bmp4* cKO mice. Thus the current series of compound genetic studies suggest that *Bmp4* is a major gene and the presence of genetic interaction between *Bmp4* and *Bmp7* for the caudal body formation. Taken together, *Bmp4* cKO mouse is a useful model to elucidate not only pathogenesis of sirenomelia but also to understand the developmental mechanisms of coordinated caudal embryonic formation.

## Materials and Methods

### Embryo collections of conditional mutant mice

The *Bmp4^flox/flo^* ^x^ and *Bmp7^flox/flox^* conditional mutant alleles employed in this study were described previously [57], [58]. The *Is1l*-Cre, *Isl1*-mER-Cre-mER and *Hoxa3*-Cre, *R26R-LacZ* indicator mice strains were generated as described previously [24], [27], [28], [31]. For embryonic sampling, pregnant females were sacrificed between E8.5 to E18.5 and the embryos were examined. All animal experiments were approved by the animal study committee of Wakayama Medical University and Kumamoto University School of Medicine (*Permit Number:* 519 in Wakayama Medical University and A23-066, A23-069 and A23-073 in Kumamoto University). The tamoxifen (TM)-inducible Cre recombinase system removes the floxed sequence from the target genome [59]. TM (Sigma, St Louis, MO, USA) was dissolved in sesame oil at 10 mg/ml. 4 mg or 2 mg of TM per 40 g body weight was used to treat the pregnant mice. Under these conditions, no overt teratologic effects are observed in the hindlimb and pelvic/urogenital organs [11].

### Histology, immunohistochemistry and X-gal staining analysis and skeletal preparation

The embryonic specimens were fixed overnight in 4% paraformaldehyde (PFA)/PBS, dehydrated in methanol and embedded in paraffin. 6 µm serial sections were prepared for Hematoxylin and Eosin (HE) staining and immunohistochemistry.

The tissue sections were stained with primary antibodies to pSMAD1/5/8 (Cell signaling) [60]. Immunostaining was visualized using DAB against primary antibodies (WAKO). X-gal staining for the detection of *Isl1*-expressing cells was performed as previously described [11]. Bones and cartilage of E18.5 fetuses were stained with alizarin red and alcian blue as previously described [61].

### In situ hybridization for gene expression analysis

Section in situ hybridization analysis was performed on 8 µm paraffin sections as previously described [62]. Whole-mount in situ hybridization was performed by standard procedures. For in situ hybridization, the following riboprobe templates were used: *Bmp4*, *Shh*, *Gli1*, *Isl1*, *Tbx4*, *Tbx5*, *Fgf8*, *Wnt5a* and *Lmx1b* (kindly provided by B.L. Hogan, N. Ueno, C. Shukunami, C.C. Hui, S. Evans, T. Ogura, TP. Yamaguchi, and RL. Johnson).

## Supporting Information

Figure S1 **Expression of** ***Tbx4*** **and** ***Tbx5*** **in** ***Shh*** **KO mice.** (A) SEM (scanning electron microscopy) image of the mouse cloacal region at E10.5. The square indicates the aPCM region. (B, C) Whole-mount ventral view of LacZ-stained embryos of caudal body region. *Isl1*-expressing cells between E8.5 and E9.5 are located in the posterior mesenchyme of the hindlimb bud and in the aPCM region (B, C). The brackets indicate the aPCM region. (D–G) ISH for the expression of the aPCM marker genes such as *Tbx4* and *Tbx5*. The expression of these genes remains in *Shh* KO mice at E10.5 (arrow in E and G). c,cloaca; t, tail; hl, hindlimb bud.
(TIF)

## References

[1] Garrido-Allepuz C, Haro E, Gonzalez-Lamuno D, Martinez-Frias ML, Bertocchini F, et al (2011) A clinical and experimental overview of sirenomelia: insight into the mechanisms of congenital limb malformations. Dis Model Mech 4: 289–299. 21504909 10.1242/dmm.007732 PMC3097451

[2] Kallen B, Castilla EE, Lancaster PA, Mutchinick O, Knudsen LB, et al (1992) The cyclops and the mermaid: an epidemiological study of two types of rare malformation. J Med Genet 29: 30–35. 1552541 10.1136/jmg.29.1.30 PMC1015818

[3] Stevenson RE, Jones KL, Phelan MC, Jones MC, Barr M, et al (1986) Vascular steal: the pathogenetic mechanism producing sirenomelia and associated defects of the viscera and soft tissues. Pediatrics 78: 451–457. 3748679

[4] Kallen B, Winberg J (1974) Caudal mesoderm pattern of anomalies: from renal agenesis to sirenomelia. Teratology 9: 99–111. 4812361 10.1002/tera.1420090113

[5] Opitz JM, Zanni G, Reynolds JF Jr, Gilbert-Barness E (2002) Defects of blastogenesis. Am J Med Genet 115: 269–286. 12503120 10.1002/ajmg.10983

[6] de Santa Barbara P, Roberts DJ (2002) Tail gut endoderm and gut/genitourinary/tail development: a new tissue-specific role for Hoxa13. Development 129: 551–561. 11830557 10.1242/dev.129.3.551 PMC2435615

[7] Kimmel SG, Mo R, Hui CC, Kim PC (2000) New mouse models of congenital anorectal malformations. J Pediatr Surg 35: 227–230 discussion 230-231. 10693670 10.1016/s0022-3468(00)90014-9

[8] Penington EC, Hutson JM (2003) The absence of lateral fusion in cloacal partition. J Pediatr Surg 38: 1287–1295. 14523808 10.1016/s0022-3468(03)00384-1

[9] Yamada G, Satoh Y, Baskin LS, Cunha GR (2003) Cellular and molecular mechanisms of development of the external genitalia. Differentiation 71: 445–460. 14641326 10.1046/j.1432-0436.2003.7108001.x

[10] Yamada G, Suzuki K, Haraguchi R, Miyagawa S, Satoh Y, et al (2006) Molecular genetic cascades for external genitalia formation: an emerging organogenesis program. Dev Dyn 235: 1738–1752. 16598715 10.1002/dvdy.20807

[11] Haraguchi R, Motoyama J, Sasaki H, Satoh Y, Miyagawa S, et al (2007) Molecular analysis of coordinated bladder and urogenital organ formation by Hedgehog signaling. Development 134: 525–533. 17202190 10.1242/dev.02736

[12] Haraguchi R, Matsumaru D, Nakagata N, Miyagawa S, Suzuki K, et al (2012) The Hedgehog Signal Induced Modulation of Bone Morphogenetic Protein Signaling: An Essential Signaling Relay for Urinary Tract Morphogenesis. PLoS ONE In press. 10.1371/journal.pone.0042245 PMC3408458 22860096

[13] Mildenberger H, Kluth D, Dziuba M (1988) Embryology of bladder exstrophy. J Pediatr Surg 23: 166–170. 3343652 10.1016/s0022-3468(88)80150-7

[14] Haraguchi R, Mo R, Hui CC, Motoyama J, Makino S, et al (2001) Unique functions of Sonic hedgehog signaling during external genitalia development. Development 128: 4241–4250. 11684660 10.1242/dev.128.21.4241

[15] Benazet JD, Zeller R (2009) Vertebrate limb development: moving from classical morphogen gradients to an integrated 4-dimensional patterning system. Cold Spring Harb Perspect Biol 1: a001339. 20066096 10.1101/cshperspect.a001339 PMC2773624

[16] Arnold SJ, Robertson EJ (2009) Making a commitment: cell lineage allocation and axis patterning in the early mouse embryo. Nat Rev Mol Cell Biol 10: 91–103. 19129791 10.1038/nrm2618

[17] Morrisey EE, Hogan BL (2010) Preparing for the first breath: genetic and cellular mechanisms in lung development. Dev Cell 18: 8–23. 20152174 10.1016/j.devcel.2009.12.010 PMC3736813

[18] Wang J, Greene SB, Martin JF (2011) BMP signaling in congenital heart disease: new developments and future directions. Birth Defects Res A Clin Mol Terato 91: 441–448. 10.1002/bdra.20785 PMC3124406 21384533

[19] Warburton D, Bellusci S (2004) The molecular genetics of lung morphogenesis and injury repair. Paediatr Respir Rev 5: Suppl A: S283–287. 14980285 10.1016/s1526-0542(04)90052-8

[20] Dudley AT, Lyons KM, Robertson EJ (1995) A requirement for bone morphogenetic protein-7 during development of the mammalian kidney and eye. Genes Dev 9: 2795–2807. 7590254 10.1101/gad.9.22.2795

[21] Luo G, Hofmann C, Bronckers AL, Sohocki M, Bradley A, et al (1995) BMP-7 is an inducer of nephrogenesis, and is also required for eye development and skeletal patterning. Genes Dev 9: 2808–2820. 7590255 10.1101/gad.9.22.2808

[22] Zakin L, Reversade B, Kuroda H, Lyons KM, De Robertis EM (2005) Sirenomelia in Bmp7 and Tsg compound mutant mice: requirement for Bmp signaling in the development of ventral posterior mesoderm. Development 132: 2489–2499. 15843411 10.1242/dev.01822

[23] Winnier G, Blessing M, Labosky PA, Hogan BL (1995) Bone morphogenetic protein-4 is required for mesoderm formation and patterning in the mouse. Genes Dev 9: 2105–2116. 7657163 10.1101/gad.9.17.2105

[24] Yang L, Cai CL, Lin L, Qyang Y, Chung C, et al (2006) Isl1Cre reveals a common Bmp pathway in heart and limb development. Development 133: 1575–1585. 16556916 10.1242/dev.02322 PMC5576437

[25] Kawakami Y, Marti M, Kawakami H, Itou J, Quach T, et al (2011) Islet1-mediated activation of the beta-catenin pathway is necessary for hindlimb initiation in mice. Development 138: 4465–4473. 21937598 10.1242/dev.065359 PMC3177316

[26] Stocker JT, Heifetz SA (1987) Sirenomelia. A morphological study of 33 cases and review of the literature. Perspect Pediatr Pathol 10: 7–50. 3588246

[27] Soriano P (1999) Generalized lacZ expression with the ROSA26 Cre reporter strain. Nat Genet 21: 70–71. 9916792 10.1038/5007

[28] Laugwitz KL, Moretti A, Lam J, Gruber P, Chen Y, et al (2005) Postnatal isl1+ cardioblasts enter fully differentiated cardiomyocyte lineages. Nature 433: 647–653. 15703750 10.1038/nature03215 PMC5578466

[29] Park EJ, Sun X, Nichol P, Saijoh Y, Martin JF, et al (2008) System for tamoxifen-inducible expression of cre-recombinase from the Foxa2 locus in mice. Dev Dyn 237: 447–453. 18161057 10.1002/dvdy.21415

[30] Zhu J, Nakamura E, Nguyen MT, Bao X, Akiyama H, et al (2008) Uncoupling Sonic hedgehog control of pattern and expansion of the developing limb bud. Dev Cell 14: 624–632. 18410737 10.1016/j.devcel.2008.01.008 PMC8284562

[31] Macatee TL, Hammond BP, Arenkiel BR, Francis L, Frank DU, et al (2003) Ablation of specific expression domains reveals discrete functions of ectoderm- and endoderm-derived FGF8 during cardiovascular and pharyngeal development. Development 130: 6361–6374. 14623825 10.1242/dev.00850 PMC1876660

[32] Goncalves A, Zeller R (2011) Genetic analysis reveals an unexpected role of BMP7 in initiation of ureteric bud outgrowth in mouse embryos. PLoS ONE 6: e19370. 21552539 10.1371/journal.pone.0019370 PMC3084290

[33] Solloway MJ, Robertson EJ (1999) Early embryonic lethality in Bmp5;Bmp7 double mutant mice suggests functional redundancy within the 60A subgroup. Development 126: 1753–1768. 10079236 10.1242/dev.126.8.1753

[34] Bandyopadhyay A, Tsuji K, Cox K, Harfe BD, Rosen V, et al (2006) Genetic analysis of the roles of BMP2, BMP4, and BMP7 in limb patterning and skeletogenesis. PLoS Genet 2: e216. 17194222 10.1371/journal.pgen.0020216 PMC1713256

[35] Bonilla-Claudio M, Wang J, Bai Y, Klysik E, Selever J, et al (2012) Bmp signaling regulates a dose-dependent transcriptional program to control facial skeletal development. Development 139: 709–719. 22219353 10.1242/dev.073197 PMC3265059

[36] Moon AM, Capecchi MR (2000) Fgf8 is required for outgrowth and patterning of the limbs. Nat Genet 26: 455–459. 11101845 10.1038/82601 PMC2001274

[37] Benazet JD, Bischofberger M, Tiecke E, Goncalves A, Martin JF, et al (2009) A self-regulatory system of interlinked signaling feedback loops controls mouse limb patterning. Science 323: 1050–1053. 19229034 10.1126/science.1168755

[38] Zakany J, Zacchetti G, Duboule D (2007) Interactions between HOXD and Gli3 genes control the limb apical ectodermal ridge via Fgf10. Dev Biol 306: 883–893. 17467687 10.1016/j.ydbio.2007.03.517

[39] Selever J, Liu W, Lu MF, Behringer RR, Martin JF (2004) Bmp4 in limb bud mesoderm regulates digit pattern by controlling AER development. Dev Biol 276: 268–279. 15581864 10.1016/j.ydbio.2004.08.024

[40] Yamaguchi TP, Bradley A, McMahon AP, Jones S (1999) A Wnt5a pathway underlies outgrowth of multiple structures in the vertebrate embryo. Development 126: 1211–1223. 10021340 10.1242/dev.126.6.1211

[41] Zeller R, Lopez-Rios J, Zuniga A (2009) Vertebrate limb bud development: moving towards integrative analysis of organogenesis. Nat Rev Genet 10: 845–858. 19920852 10.1038/nrg2681

[42] Zakany J, Kmita M, Duboule D (2004) A dual role for Hox genes in limb anterior-posterior asymmetry. Science 304: 1669–1672. 15192229 10.1126/science.1096049

[43] Bastida MF, Sheth R, Ros MA (2009) A BMP-Shh negative-feedback loop restricts Shh expression during limb development. Development 136: 3779–3789. 19855020 10.1242/dev.036418

[44] Browne M, Fitchev P, Adley B, Crawford SE (2004) Sirenomelia with an angiomatous lumbosacral myelocystocele in a full-term infant. J Perinatol 24: 329–331. 15116133 10.1038/sj.jp.7211085

[45] Taori KB, Mitra K, Ghonga NP, Gandhi RO, Mammen T, et al (2002) Sirenomelia sequence (mermaid): Report of three cases. Indian J Radiol Imaging 12: 399–401.

[46] Chen H, Lun Y, Ovchinnikov D, Kokubo H, Oberg KC, et al (1998) Limb and kidney defects in Lmx1b mutant mice suggest an involvement of LMX1B in human nail patella syndrome. Nat Genet 19: 51–55. 9590288 10.1038/ng0598-51

[47] Loomis CA, Harris E, Michaud J, Wurst W, Hanks M, et al (1996) The mouse Engrailed-1 gene and ventral limb patterning. Nature 382: 360–363. 8684466 10.1038/382360a0

[48] Naiche LA, Papaioannou VE (2007) Tbx4 is not required for hindlimb identity or post-bud hindlimb outgrowth. Development 134: 93–103. 17164415 10.1242/dev.02712

[49] Ouimette JF, Jolin ML, L'Honore A, Gifuni A, Drouin J (2010) Divergent transcriptional activities determine limb identity. Nat Commun 1: 35. 20975709 10.1038/ncomms1036 PMC3046407

[50] Naiche LA, Arora R, Kania A, Lewandoski M, Papaioannou VE (2011) Identity and fate of Tbx4-expressing cells reveal developmental cell fate decisions in the allantois, limb, and external genitalia. Dev Dyn 240: 2290–2300. 21932311 10.1002/dvdy.22731 PMC3180884

[51] Ohta S, Suzuki K, Tachibana K, Tanaka H, Yamada G (2007) Cessation of gastrulation is mediated by suppression of epithelial-mesenchymal transition at the ventral ectodermal ridge. Development 134: 4315–4324. 18003744 10.1242/dev.008151

[52] Barr M Jr (1988) Comments on “Origin of Abnormality in a Human Simelian Foetus as Elucidated by Our Knowledge of Vertebrate Development”. Teratology 38: 487–491. 3238607 10.1002/tera.1420380513

[53] O'Rahilly R, Muller F (1989) Interpretation of some median anomalies as illustrated by cyclopia and symmelia. Teratology 40: 409–421. 2623629 10.1002/tera.1420400502

[54] Chiang C, Litingtung Y, Harris MP, Simandl BK, Li Y, et al (2001) Manifestation of the limb prepattern: limb development in the absence of sonic hedgehog function. Dev Biol 236: 421–435. 11476582 10.1006/dbio.2001.0346

[55] Yuan S, Schoenwolf GC (2000) Islet-1 marks the early heart rudiments and is asymmetrically expressed during early rotation of the foregut in the chick embryo. Anat Rec 260: 204–207. 10993956 10.1002/1097-0185(20001001)260:2<204::AID-AR90>3.0.CO;2-5

[56] Chevallier A, Kieny M, Mauger A (1977) Limb-somite relationship: origin of the limb musculature. J Embryol Exp Morphol 41: 245–258. 591873

[57] Chang W, Lin Z, Kulessa H, Hebert J, Hogan BL, et al (2008) Bmp4 is essential for the formation of the vestibular apparatus that detects angular head movements. PLoS Genet 4: e1000050. 18404215 10.1371/journal.pgen.1000050 PMC2274953

[58] Zouvelou V, Passa O, Segklia K, Tsalavos S, Valenzuela DM, et al (2009) Generation and functional characterization of mice with a conditional BMP7 allele. Int J Dev Biol 53: 597–603. 19247966 10.1387/ijdb.082648vz

[59] Feil R, Wagner J, Metzger D, Chambon P (1997) Regulation of Cre recombinase activity by mutated estrogen receptor ligand-binding domains. Biochem Biophys Res Commun 237: 752–757. 9299439 10.1006/bbrc.1997.7124

[60] Omori A, Harada M, Ohta S, Villacorte M, Sugimura Y, et al (2011) Epithelial Bmp (Bone morphogenetic protein) signaling for bulbourethral gland development: a mouse model for congenital cystic dilation. Congenit Anom (Kyoto) 51: 102–109. 21848994 10.1111/j.1741-4520.2011.00318.x

[61] Harada M, Murakami H, Okawa A, Okimoto N, Hiraoka S, et al (2009) FGF9 monomer-dimer equilibrium regulates extracellular matrix affinity and tissue diffusion. Nat Genet 41: 289–298. 19219044 10.1038/ng.316 PMC2676118

[62] Suzuki K, Haraguchi R, Ogata T, Barbieri O, Alegria O, et al (2008) Abnormal urethra formation in mouse models of split-hand/split-foot malformation type 1 and type 4. Eur J Hum Genet 16: 36–44. 17878916 10.1038/sj.ejhg.5201925

