# Supplementary figures and images for: Reduced BMP Signaling Results in Hindlimb Fusion with Lethal Pelvic/Urogenital Organ Aplasia: A New Mouse Model of Sirenomelia
Source: PLoS One. 2012 Sep 17;7(9):e43453. doi: 10.1371/journal.pone.0043453 (PMC3444444; doi:10.1371/journal.pone.0043453)

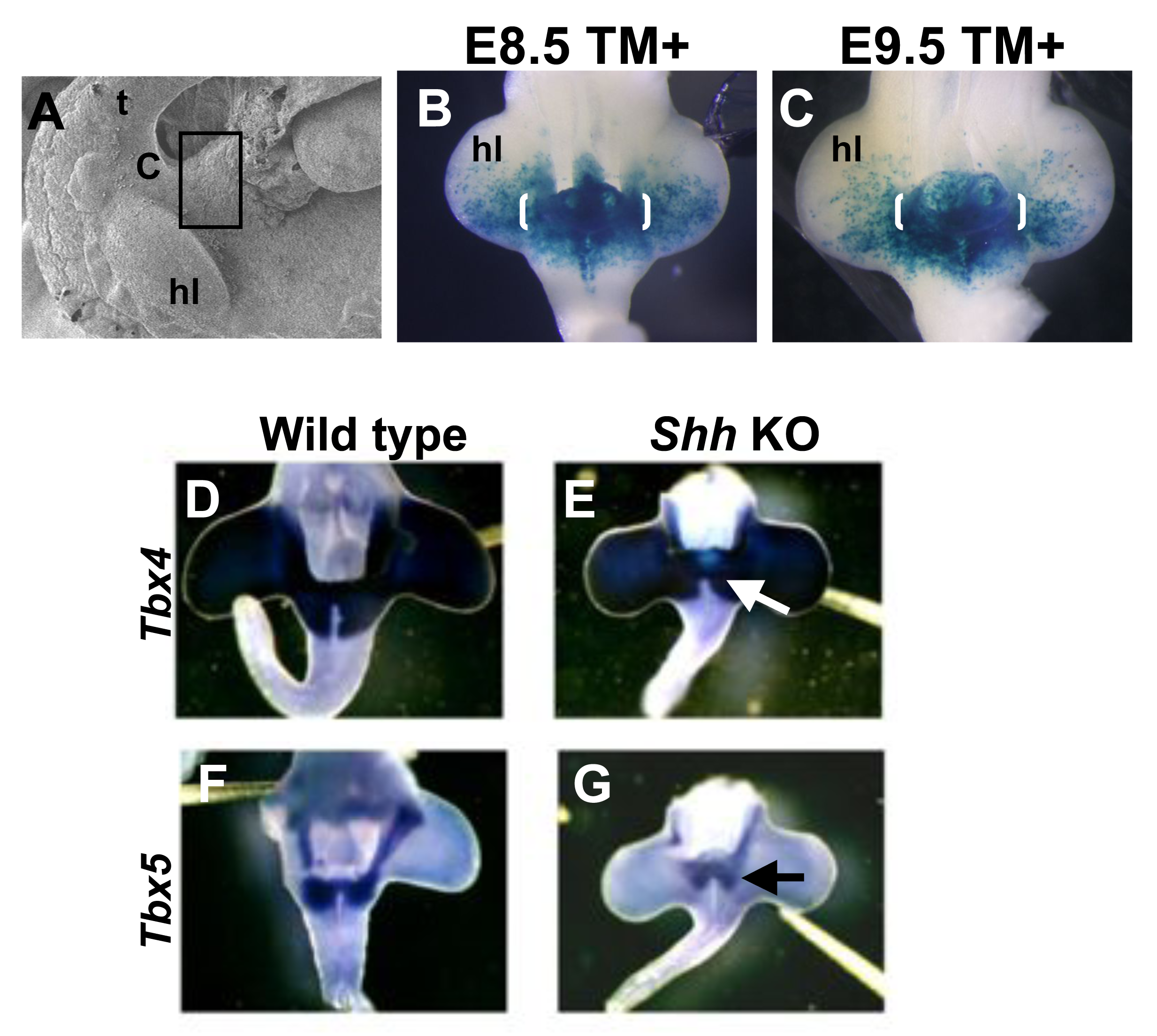

Supplement: Figure S1 — Expression of Tbx4 and Tbx5 in Shh KO mice. (A) SEM (scanning electron microscopy) image of the mouse cloacal region at E10.5. The square indicates the aPCM region. (B, C) Whole-mount ventral view of LacZ-stained embryos of caudal body region. Isl1-expressing cells between E8.5 and E9.5 are located in the posterior mesenchyme of the hindlimb bud and in the aPCM region (B, C). The brackets indicate the aPCM region. (D–G) ISH for the expression of the aPCM marker genes such as Tbx4 and Tbx5. The expression of these genes remains in Shh KO mice at E10.5 (arrow in E and G). c,cloaca; t, tail; hl, hindlimb bud. (TIF) [file pone.0043453.s001.tif]
